# MIND4OLIGOS: Determining the Monoisotopic Mass of Oligonucleotides Observed in High-Resolution Mass Spectrometry

**DOI:** 10.1021/acs.analchem.3c04351

**Published:** 2024-05-28

**Authors:** Piotr Prostko, Piotr Radziński, Michał Ciach, Youzhong Liu, Michał Startek, Frederik Lermyte, Thomas De Vijlder, Anna Gambin, Simon Appeltans, Dirk Valkenborg

**Affiliations:** † Faculty of Science, Data Science Institute, Interuniversity Institute for Biostatistics and statistical Bioinformatics, Center for Statistics, Hasselt University, Agoralaan, Diepenbeek BE 3500, Belgium; ‡ Institute of Informatics, University of Warsaw, Banacha 2, Warszawa PL 02-097, Poland; § Johnson & Johnson Innovative Medicine, Therapeutics Development & Supply, Turnhoutseweg 30, Beerse BE 2340, Belgium; ∥ University Medical Center of the Johannes Gutenberg University Mainz, Institute of Immunology, Mainz, Rheinland-Pfalz 55131, Germany; ⊥ Department of Chemistry, Technical University of Darmstadt, Darmstadt, Hessen 64289, Germany; # Centre for Synthetic Biology, Technical University of Darmstadt, Darmstadt, Hessen 64289, Germany

## Abstract

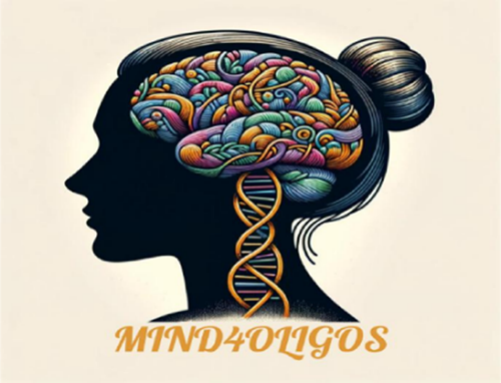

Oligonucleotide therapeutics have emerged as an important class of drugs offering targeted therapeutic strategies that complement traditional modalities, such as monoclonal antibodies and small molecules. Their unique ability to precisely modulate gene expression makes them vital for addressing previously undruggable targets. A critical aspect of developing these therapies is characterizing their molecular composition accurately. This includes determining the monoisotopic mass of oligonucleotides, which is essential for identifying impurities, degradants, and modifications that can affect the drug efficacy and safety. Mass spectrometry (MS) plays a pivotal role in this process, yet the accurate interpretation of complex mass spectra remains challenging, especially for large molecules, where the monoisotopic peak is often undetectable. To address this issue, we have adapted the MIND algorithm, originally developed for top-down proteomics, for use with oligonucleotide data. This adaptation allows for the prediction of monoisotopic mass from the more readily detectable, most-abundant peak mass, enhancing the ability to annotate complex spectra of oligonucleotides. Our comprehensive validation of this modified algorithm on both in silico and real-world oligonucleotide data sets has demonstrated its effectiveness and reliability. To facilitate wider adoption of this advanced analytical technique, we have encapsulated the enhanced MIND algorithm in a user-friendly Shiny application. This online platform simplifies the process of annotating complex oligonucleotide spectra, making advanced mass spectrometry analysis accessible to researchers and drug developers. The application is available at https://valkenborg-lab.shinyapps.io/mind4oligos/.

## 1 Introduction

The essential function of nucleic acids in the governance of cellular processes in the human body makes them perhaps one of the most extensively researched biopolymers. As such, many analytical platforms and techniques have gradually become available for investigating DNA/RNA-related research problems in various contexts. After developing soft ionization methods such as matrix-assisted laser desorption ionization (MALDI) and electrospray ionization (ESI), the analysis of nucleic acids and oligonucleotides with mass spectrometry (MS) has turned into a viable option.^1−5^

The increasing interest in the MS-based analysis of nucleic acids and oligonucleotides was, and still is, particularly noticeable in the pharmaceutical industry. Pourshaian et al. provide an overview of the tasks typically performed in MS-based analyses of therapeutic oligonucleotides.^5^ Sequence confirmation and the characterization of process impurities and degradation products in drug substances and finished drug products are examples of such tasks. In fact, they are also a part of the ‘identity testing’ activities required by regulatory bodies before releasing the drug substance or drug product into clinics or the market.^6^ An example of clinically and commercially successful oligonucleotide from recent years is nusinersen (Spinraza)—a modified antisense oligonucleotide (ASO) therapy for spinal muscular atrophy^7^ Besides ASOs, other types of synthetic oligonucleotides are used in drug development: aptamers, micro-RNAs, small interfering RNAs (siRNAs), and clustered regularly interspaced short palindromic repeats (CRISPR). Each class has a different mechanism of action, physiochemical attributes, and metabolic profile. Various modification strategies applied to the phosphate backbone, base, and sugar have been developed to enhance a drug’s in vivo stability and integrity and improve a drug’s distribution and potency. These chemical alterations contribute to considerable structural complexity of therapeutic oligonucleotides, triggering an increased demand for the MS-based characterization.

Technological advancements in mass spectrometry and trends instigated by pharmaceutical companies have inclined researchers toward exploring new applications in nucleic acid research. This progress, however, has taken place at a much slower pace than the quickly evolving MS-based proteomics. A possible contributing factor to this difference is the scarcity of customized bioinformatics algorithms and software to process and interpret vast amounts of spectral information generated by studying oligonucleotides.^8−10^

It, therefore, becomes more evident that together with the increasing diversity of MS applications, the need for novel bioinformatics will only grow over time. A feasible approach to identify analytes with MS involves comparing the isotope distribution observed in an experimental spectrum and the one theoretically computed based on the known molecular formula of the molecule of interest.^11−13^ However, doing so in an automated way becomes impossible if one does not know the molecular formula. To overcome this limitation, Agten et al.^14^ recently derived a method that models isotope information and returns a prediction of the aggregated isotope distribution of an average oligonucleotide. They have shown that the proposed model is straightforward to use and accurate. However, the model requires information about the monoisotopic mass as an input. Unfortunately, the abundance of the monoisotopic variant of large molecules such as DNA- or RNA-based therapeutics is frequently below the detection level of mass spectrometers, preventing an adequate use of the model of Agten et al. To overcome this issue, one could envisage the use of the MIND algorithm^15^ that predicts the monoisotopic mass based on the most-abundant peak in the experimental spectra of proteins. The problem is that MIND was developed based on the composition of proteins and does not work as published for oligonucleotides.

Despite the progress in proteomics, a gap remains in directly transferring monoisotopic mass detection methods to DNA and RNA analyses without significant recalibration.^16−18^ This work presents a novel adaptation of the MIND algorithm for oligonucleotide mass spectrometry, aptly named MIND4OLIGOS. This approach seamlessly bridges this gap, offering a targeted solution for accurately characterizing DNA and RNA molecules. MIND4OLIGOS has undergone extensive validation on both simulated and real DNA data sets, demonstrating its accuracy and reliability. While our focus primarily lies on DNA, the model’s efficacy extends to RNA oligonucleotides, as detailed in the Supporting Information. The models are optimized for mass ranges specific to DNA (1463.242–30303.87 Da) and RNA (1543.22–31086.31 Da), with caution against extrapolation beyond these intervals.

## 2 Materials and Methods

### 2.1 Experimental Procedures

To validate our proposed prediction method on real-life data, we examined oligonucleotides introduced in Agten et al.^14^ The molecules were acquired from Integrated DNA Technologies (Leuven, Belgium). These oligonucleotides for the proof-of-concept study were analyzed via liquid chromatography–mass spectrometry (LC-MS) by injecting 10.5 μL of a ca. 0.5 mg/mL aqueous solution on an Agilent 1290 UPLC (Waters Inc., Antwerp, Belgium) coupled to a timsTOF Pro mass spectrometer (Bruker, GmbH Germany). Chromatographic separations were performed using an Acquity BEH 300 C18 column (150 mm × 2.1 mm, 1.7 μm particle size) (Waters Inc.). The column heater was kept at 75 °C, and the flow rate was 0.25 mL/min; mobile phase A consisted of 7 mM triethylamine and 60 mM 1,1,1,3,3,3-hexafluoro-2-propanol (HFIP) in water, and mobile phase B was a methanol–acetonitrile mixture (50:730, v/v). The gradient elution consisted of a linear gradient of 0–65% of eluent B in 10 min, increased to 100% eluent B for 3 min, followed by a washing step of 1 min at 100% eluent B. High-resolution accurate mass data were acquired in negative ion mode using an electrospray ionization source using a mass range of *m*/*z* 500 to *m*/*z* 2000 with a spectral rate of 2.0 Hz. The following source conditions were applied: a capillary voltage of 2.5 kV, a dry temperature of 200 °C, and a dry gas flow of 8 L/min.

MSConvert GUI^19^ was applied to transform the raw vendor spectral files to mzXML format. Further necessary processing steps were performed automatically in R programming language with an in-house script called OligoDistiller (https://github.com/daniellyz/OligoDistiller). The elution and charge-state range presented in Table 1 were determined by manual inspection to ensure good spectral quality and integrity. To extract isotopic envelopes of one oligonucleotide from the LC-MS data, we first selected all MS scans within the elution ranges. Subsequently, the charge state and neutral molecular weight (NMW) of each mass peak in the selected scans were computed by OligoDistiller. Isotopic envelope replicates of the same oligonucleotide were then collected from the scans according to the charge state and the NMW range specified in Table 1. The NMW range was defined between the monoisotopic molecular weight (MMW) of an oligonucleotide and MMW + 15 Da.

**Table 1:** Three DNA Strands Originally Measured for the Proof-of-Concept Study of the Average Isotopic Distribution Prediction Method of Agten et al. Were Remeasured for This Study to Validate MIND4OLIGOS

name	DNA_SHORT1	DNA_SHORT2	DNA_LONG
type	DNA	DNA	DNA
sequence	GCC ACA TAT GAG AGT GGA TTT GTC ATT	GGT GCC CCA GAA TCT CTC AGC CT	GAG ATC TCT GCT TCT GAT GGC TCT CTG GTT ACT GCC AGT TGA ATC TG
molecular formula	C_266_H_334_N_100_O_162_P_26_	C_221_H_282_N_82_O_137_P_22_	C_459_H_582_N_162_O_290_P_46_
monoisotopic mass (Da)	8325.41	6957.18	14,426.37
NMW range (Da)	8325–8340	6957–6972	14,426–14,441
charge-state range	8–12	6–10	10–20
elution ranges	2.52–2.65 min (15 scans)	2.43–2.50 min (9 scans)	2.67–2.90 min (27 scans)
# of extracted isotopic envelopes	75	45	270

### 2.2 In Silico-Generated Data

The data required for recalibrating the MIND algorithm were taken from Agten et al.^14^ The data consist of a complete set of all combinations of DNA nucleotides ranging from 5 to 92 DNA bases (adenine, cytosine, guanine, and thymine with a 2′-deoxyribose phosphodiester backbone). Tables S1 and S2 in the Supporting Information of Agten et al.^14^ present necessary information for the computation of the molecular formula of the oligonucleotides, bearing in mind that the addition of each nucleotide implies the loss of one water molecule to form the phosphodiester link. The BRAIN^20,21^ method was employed to obtain the intensities and exact center masses of the 30 first isotopes in the theoretically expected aggregated isotopic distribution based on the atomic compositions of our DNA oligonucleotides.

The procedure described above resulted in 3,321,890 DNA oligonucleotides of length between 5 and 92 bases and a monoisotopic molecular weight encompassing 1463.24 to 30,290.84 Da. The lightest oligonucleotide is (dCMP)_5_, while the heaviest is the (dGMP)_92_ molecule. Contrary to amino acids, the theoretical DNA nucleotide combinations (not permutations) give rise to unique atomic compositions and consequently mass values. Furthermore, Figure S1 in the Supporting Information shows that the ratio of monoisotopic and most-abundant variant intensities falls below 5% for molecules heavier than 11,181 Da.

Before any modeling efforts, 10,000 DNA oligonucleotides were randomly selected from our in silico-generated data. This process resulted in a validation set, comprising the chosen 10,000 molecules, and a training set, consisting of all remaining molecules. Figure S2 confirms that the sampling was conducted correctly, and the mass distribution of the test data molecules is comparable to that of the training set molecules.

### 2.3 Modifications of the MIND Algorithm

**Figure 1:**
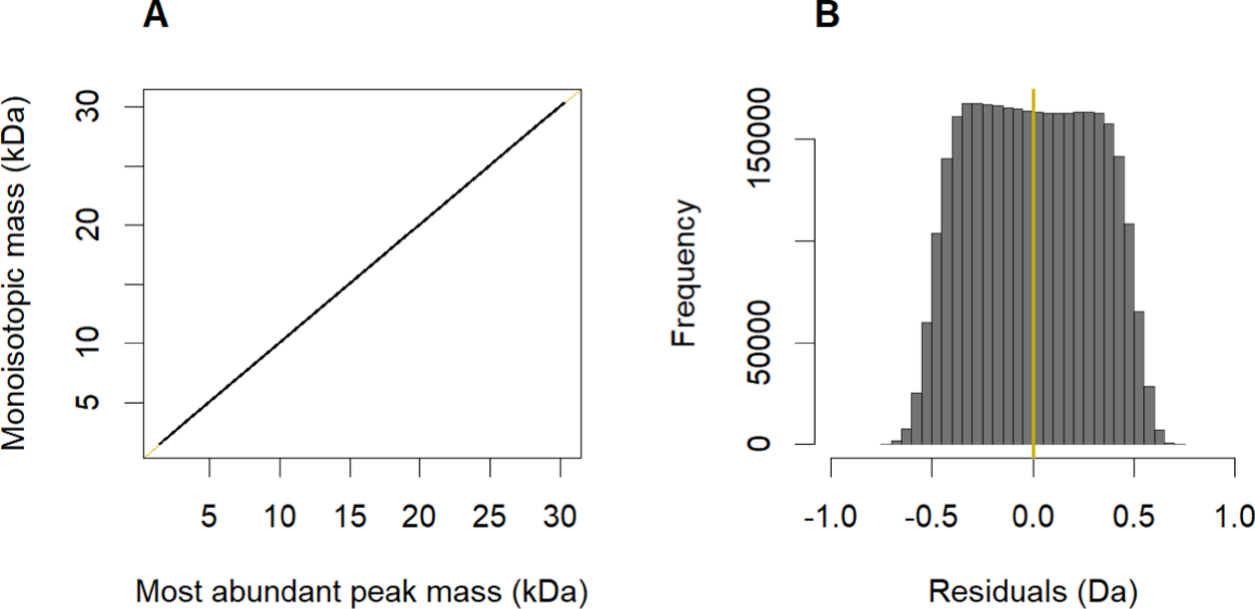
(A) Monoisotopic masses of DNA oligonucleotides from the training data set (*n* = 3,311,890) are linearly related to their most-abundant masses. (B) Width of the resulting error distribution is too large to consider this linear model valuable in real-world applications.

Visualizing all of the DNA molecules from the training set revealed a linear statistical relationship between their monoisotopic and most-abundant ion masses (Figure 1A), which is similar to what was observed for proteins.^15^ As such, we fitted a linear model given by the equation
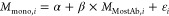
1with *i* being the index of DNA oligonucleotide in the data, *M*_mono_ the monoisotopic mass, *M*_MostAb_ the most-abundant mass value, and ε a random variable corresponding to the model error (i.e., model residual value). The ordinary least squares estimates of the intercept α̂ and slope β̂ were equal to approximately 0.6624 and 0.9995, respectively. Let *e*_*i*_ be the *i*-th realization of the ε random variable, calculated as follows: *e*_*i*_ = *M*_mono,*i*_ – (α̂ + β̂ × *M*_MostAb,*i*_). Nearly 99% of these residuals are (largely evenly) distributed between −0.58 and 0.58 Da (Figure 1B). Another way of looking at Figure 1B is to notice that a narrow mass interval reflecting the user-acceptable mass error range, for instance, [−0.05, 0.05] Da, contains only 10% of the analyzed theoretical molecules. Thus, the simple linear model alone is not sufficient for making accurate predictions based on the theoretical mass value of the most-abundant ion.

**Figure 2:**
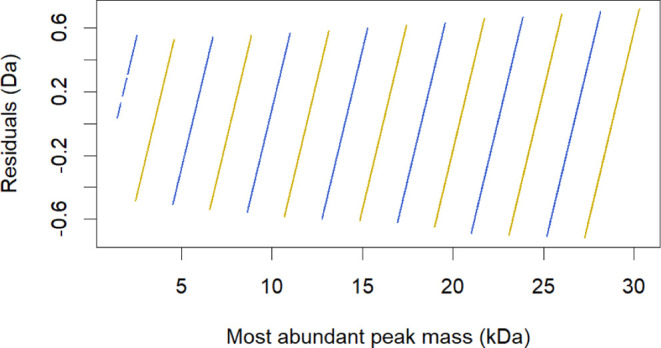
Residuals *e*_*i*_ form 14 piecewise linear patterns as a function of the most-abundant mass, allowing for further refinements and more accurate monoisotopic mass prediction. Note that lines’ domains overlap for some given masses; however, no more than two domains overlap for the same mass.

For this reason, we further investigated the possibility of modeling the *e*_*i*_ residuals to improve the accuracy of the initial linear model. Figure 2 shows that the observed patterns of the residuals as a function of the most-abundant mass can be closely captured by 14 straight lines. Note that the *x*-axis ranges of the individual line segments are not mutually exclusive as they slightly overlap. The width of the overlapping mass regions is considerably smaller than that observed in a similar graph included in the protein-focused publication of Lermyte et al.,^15^ and this fact allowed for simplifying the modeling procedure. A possible explanation of the ‘smaller’ overlap is that unmodified DNA molecules do not contain sulfur (but proteins do), which alters the shape of the isotope distribution and the relationship between monoisotopic and most-abundant peak masses. Even though the overlap is less pronounced in the oligonucleotide setting, it still occurs. The overlapping mass regions constitute around 20% of the total most-abundant mass range and comprise about 34% of all molecules and hence the need to introduce an additional analysis step for optimal line assignment.

**Figure 3:**
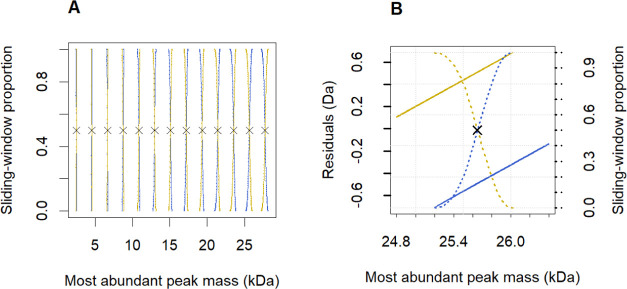
Selecting the appropriate residual line in order to determine the shift between the monoisotopic and the most-abundant masses. Line colors intentionally match the colors used in Figure 2. (A) Evolution of sliding-window proportions inside the overlapping mass regions (outside those regions, the proportions are either 0 or 1). The proportions are computed based on a 50 Da-wide sliding window that counts the number of oligonucleotides on the overlapping blue and gold residual lines. The black dots represent 50% proportion, and, as such, their *x*-axis coordinate delineates the optimal location for changing the residual lines (from upper to lower, or vice versa). (B) A close-up view into one overlapping mass region, showing the two residual lines and their corresponding sliding-window proportions. 25,651 Da is the optimal value that triggers the change from the gold to the blue residual line.

Toward this goal, we isolated the data points corresponding to each of the 14 trends and separately fitted linear functions. This step resulted in a set of estimated coefficients {*c*_*i*_, *d*_*i*_}_*i*=1,···,14_ (the intercepts and slopes are reported in Table S1 in Supporting Information) and the corresponding mass ranges or domains {[*a*_*i*_, *b*_*i*_]}_*i*=1,···,14_, for which the linear functions are valid. We will succinctly refer to this collection of 14 fitted lines as “model 2”. As already mentioned, model 2 domains {[*a*_*i*_, *b*_*i*_]}_*i*=1,···,14_ are not mutually exclusive. Therefore, we adhere to the principle of the optimal Bayes classifier and propose the following procedure to define the optimal and mutually exclusive mass ranges:

1. Move a sliding window of 50 Da across the entire mass range with steps of 10 Da and perform the proportion calculation explained in step 2.
2. At a particular mass value formed by the center of the two-sided window of 25 Da width on each side (if not possible, then a one-sided interval), compute the proportion of DNA compounds belonging to the two overlapping residual lines indicated in gold and blue in Figure 3A.
3. The optimal mass range is now defined at a mass value where the proportion between gold and blue residual lines is 50% by applying linear interpolation on the data points computed in step 2 (Figure 3B).

Essentially, this rule cuts the entire mass range into 14 nonoverlapping mass intervals given by {[*m*_1_, *m*_2_], [(*m*_*i*_, *m*_*i*+1_)]_*i*=2,···,14_}, where *m*_*i*_ is the *x*-axis value of the “optimal Bayes points” (Figure 3A). Applying the proposed heuristic reduces but does not eliminate the chance of false classifications. The misclassification error rate can be obtained by taking one minus the sliding-window proportion of the assigned residual line.

**Figure 4:**
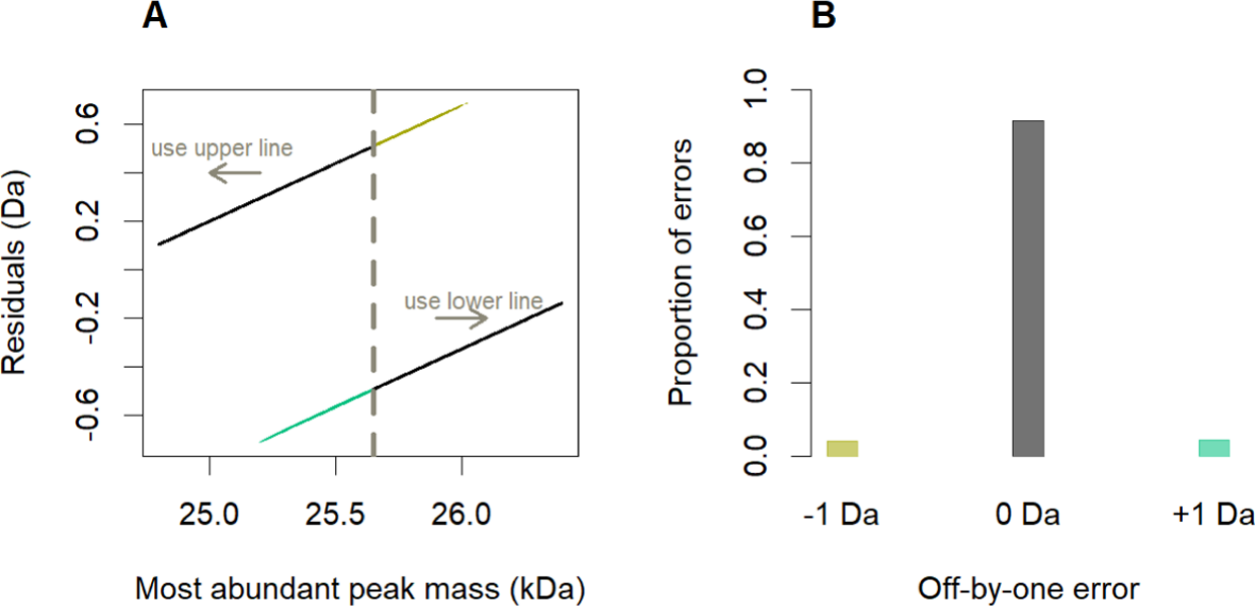
(A) Continuing the example from Figure 3B, the gray dashed line is the outcome of our classification procedure, and it designates two areas: one to the left, where the residual mass should be taken from the upper line, and the right side that makes use of the lower line. The points in black were correctly classified; all other points contribute to the “off-by-one” errors. (B) Summary of the off-by-one error in the 25,150–26,050 Da interval of the training data. The −1 Da (khaki) and +1 Da (light-blue) errors occurred at rates of 4.34 and 4.14%, respectively, and 91.52% of cases were adequately classified (gray).

The practical usage of the classification rule is visually explained in Figure 4A. The rule asserts that molecules to the left of the gray, dashed vertical line should be considered as generated by the upper residual line and those to the right side of the gray line by the bottom line. Since the intercepts of the residual lines presented in Figure 2 differ by ca. 1 Da (see Table S1 in the Supporting Information), the classification error, defined as the predicted minus true residual value, is either (approximately) equal to −1 or +1 Da. Thus, we will use the term “off-by-one error” in the remainder of this paper, and −1 and +1 Da should be interpreted more as labels than exact values. The light-blue and gold segments in Figure 4A consist of points with −1 or +1 Da errors, respectively. Figure 4B indicates that the MIND4OLIGOS algorithm can theoretically assign the correct monoisotopic mass based on the most-abundant ion mass in over 91% of the cases lying in the selected mass interval (25,150–26,050 Da). Additionally, a sliding-window proportion value, indicating the probability of making the one-by-one error, is assigned to monoisotopic mass prediction.

In summary, the proposed adaptation of the MIND method involves the evaluation of the global linear model given by eq 1 (the first parenthesis below) and predicting the residual value from an appropriate residual line determined by the sliding-window-based classification rule (the second parenthesis below)
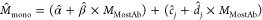
2where *M*_MostAb_ is the most-abundant peak selected in an experimental spectrum (preferably with the correction based on the experimental average and most-abundant masses explained in the paragraph below). If *M*_MostAb_ ∈ [*m*_1_, *m*_2_], then the index *j* equals 1; else, *j* ∈ {2,···,13}, and *j* satisfies *M*_MostAb_, ∈ (*m*_*j*_, *m*_*j*+1_].

#### 2.3.1 Most-Abundant Peak Selection Correction

It becomes clear now that the presented methodology strongly relies on the most-abundant peak mass. However, poor ion statistics, low-intensity signals, wide isotope distribution of larger molecules, and noisy signals, may considerably contribute to missing the most-abundant peak by ±1 Da or more, and as such an incorrect model prediction. Therefore, here, we also employed the heuristic for improved most-abundant mass selection introduced by Lermyte et al.^15^ Briefly, the heuristic is based on the observation that the difference between the intensity-weighted average mass (AM) and the true most-abundant mass (MA) of all in silico-generated DNA molecules (i.e., the training and test partitions combined) lies within the AM–MA = [0.115, 1.21] Da interval (Figure S4 in the Supporting Information). Once presented with an experimental isotopologue envelope, one should compute the difference involving the experimental average mass and the ‘naively’ chosen mode mass (i.e., the mass corresponding to the most intense signal in the isotope cluster). The underlying assumption is that factors such as noise will largely cancel out when averaged across the isotopic envelope, so that the average mass will not display any sudden “jumps” by ±1 Da. If this value exceeds the upper bound, the selected ’mode’ mass is too small, and the next peak in the series should be used as input for our prediction model. Conversely, a value below the lower bound triggers the selection of the previous peak in the isotope distribution. This procedure is repeated until the recalculated difference can be found in the interval. We additionally conducted a simulation study to assess the usefulness of the heuristic in several poor ion statistics settings. Based on the positive outcomes (see Figure S6), we can recommend employing the correction when dealing with experimental mass spectra.

It should be noted that the monoisotopic peak mass prediction is prone to two disparate types of errors: the off-by-one mistake and the invalid predictor value. The former has already been explained in Figures 3 and 4; the latter occurs due to incorrect most-abundant peak selection and is not explicitly caused by the proposed modeling approach. The most-abundant peak selection correction has some built-in tolerance against spectral mass inaccuracies in the heuristic explained above, but ‘large’ and systematic errors on the most-abundant masses will be one-to-one errors on the predicted monoisotopic masses. Therefore, it is the end-user’s responsibility to provide a well-calibrated and high-quality mass spectrum. The correction will likely fail if confronted with a mixture of two or more individual isotopic envelopes.

## 3 Results and Discussion

### 3.1 In Silico Validation

We started by examining the performance of our approach on the earlier put-aside validation set composed of 10,000 in silico-generated DNA oligos. To make this validation task more challenging, we added noise drawn from a uniform distribution with a varying support width (set to 10% of the isotope height) to the top of the theoretical isotope distributions. See the Supporting Information for more details on noise generation. Due to the generated noise, we also needed to employ the most-abundant peak mass heuristic. No noise was added to the mass values as it would be directly propagated to the monoisotopic mass prediction without revealing any insights into the prediction model’s performance.

**Figure 5:**
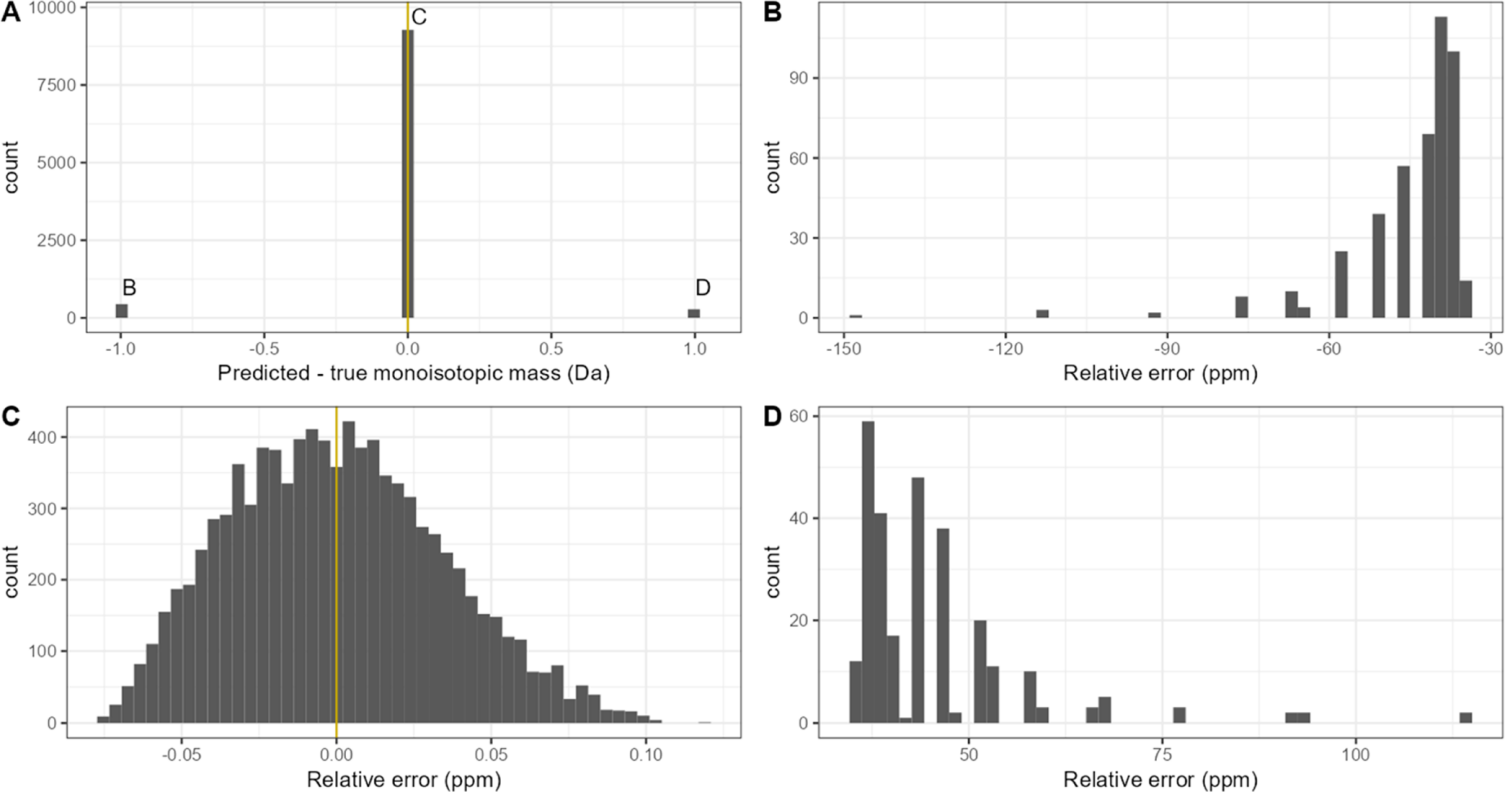
(A) In silico validation of the data concerning 10,000 in silico-generated (unmodified) DNA sequences that were set apart from the training data. The isotopic peak heights were deliberately distorted by the addition of uniform noise. The resulting distribution of the off-by-one error (i.e., predicted minus theoretical monoisotopic mass value) is as follows: 92.87% accuracy; 4.33 and 2.80% of −1 Da and +1 Da errors, respectively. (B–D) Relative prediction errors around the bars from panel (A).

Panel A of Figure 5 displays the prediction error (predicted minus true monoisotopic mass value) across the entire mass range. The classification accuracy equals 92.87%, with 4.33 and 2.80% error percentages for the −1 and +1 Da errors, respectively. The relative prediction error, expressed in ppm and split into three separate panels with a close-up view around the off-by-one errors, is presented in panels B–D of Figure 5.

### 3.2 Application of MIND4OLIGOS to High-Resolution MS Data of Oligonucleotides

#### 3.2.1 Full-Length Product Analysis

As indicated in Table 1, isotopic envelopes of the three full-length products (i.e., the intended product of oligonucleotide synthesis) were measured in separate scans under the same chromatographic peak and detected in multiple charge states. Before the validation analysis, we checked the ion statistics (i.e., total ion intensity) and the number of peaks in the extracted isotopic distributions for each molecule–charge combination (see Figure S5 in the Supporting Information). Both the ion statistics and the peak count can be regarded as adequate, and hence there is no need for filtering out any experimental data. Note that the input for MIND4OLIGOS is the molecular weight of the most-abundant ion, not the mass-to-charge ratio. The same remark applies to the input of the most-abundant mass selection heuristic described earlier.

**Figure 6:**
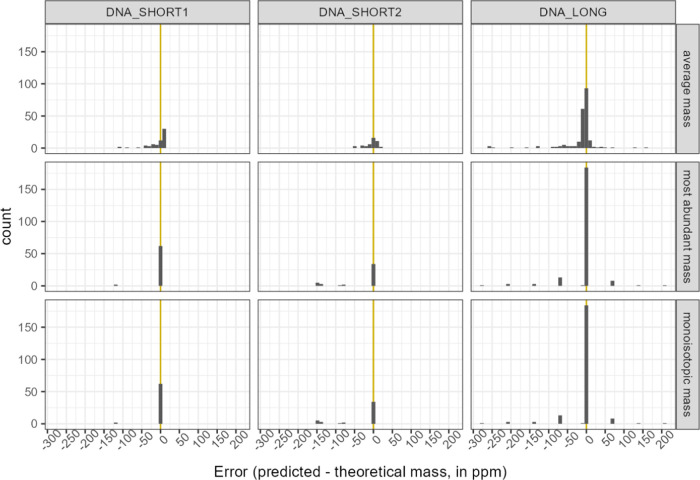
Distribution of errors in the average, most-abundant, and monoisotopic mass metrics of the three DNA compounds. It should be noted that the error in most-abundant mass is the difference between the selected (using the heuristic) and theoretical most-abundant peak mass values. DNA_SHORT1, DNA_SHORT2, and DNA_LONG were evaluated on 75, 45, and 270 envelopes, respectively. Mass scans acquired over a selected retention time range gave rise to multiple envelopes. The most-abundant ion variant was obtained by employing the correction procedure.

**Figure 7:**
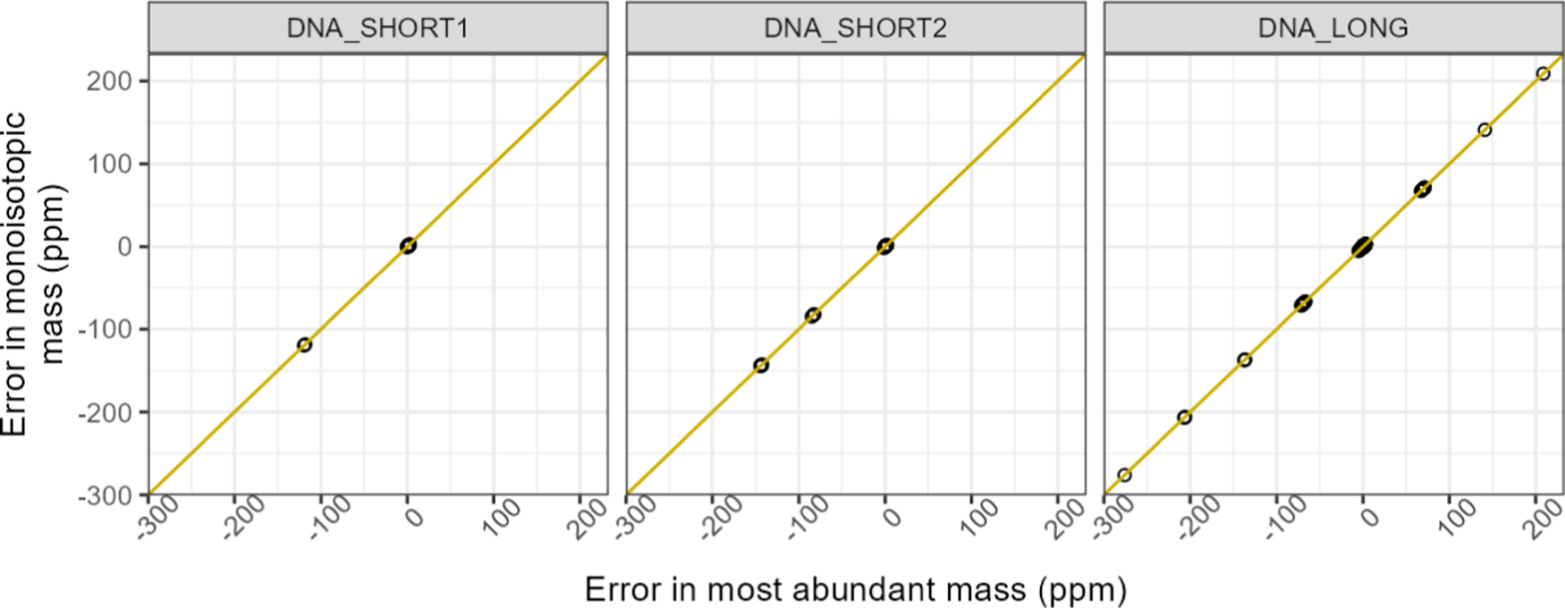
In this real-world validation data set of three oligonucleotide strands, the errors in the monoisotopic mass prediction nearly perfectly correlated with the errors in the most-abundant masses as both closely follow the gold identity line.

The results of applying our prediction method are presented in Figure 6. The first row shows the error between the experimentally determined (intensity-weighted) and the theoretical average mass. Lermyte et al.^15^ and Claesen et al.^22^ have observed that the average mass is less “precise” and less robust to various MS-related errors as a mass metric than, for instance, the most-abundant mass (second row). Our data are in line with their findings as the error in the average mass is noticeably more spread over the ppm axis compared to the error in the most-abundant mass (for exact numbers, see Table 2). One can even conjecture that using a more varying feature could worsen the prediction precision. The third row in the discussed figure presents the error in monoisotopic mass prediction, which is predominantly concentrated in the histogram bin around 0 ppm. Nevertheless, a number of isotope envelopes gave rise to deviations of a larger magnitude, i.e., tens of ppm or more. In fact, these errors correspond approximately to integer values in daltons. Figure 7 further clarifies that the substantial prediction errors occurred only when the most-abundant peak was wrongly picked from the experimental data.

**Table 2:** Central Tendency and Dispersion Measures of Errors (ppm) in the Average Mass (MA), Most-Abundant Mass (MAB), and Monoisotopic Mass (MONO)

	DNA_SHORT1			DNA_SHORT2			DNA_LONG		
	MA	MAB	MONO	MA	MAB	MONO	MA	MAB	MONO
mean	–8.005	–2.300	–2.299	–5.222	–30.455	–30.465	–14.433	–5.091	–5.078
median	3.902	1.463	1.465	–1.045	0.662	0.665	–4.632	1.613	1.629
IQR	24.629	0.728	0.728	15.523	2.588	2.589	7.295	2.032	2.032
SD	27.316	21.130	21.138	17.898	57.172	57.196	46.714	44.300	44.318
MAD	7.229	0.366	0.366	7.724	0.498	0.499	3.631	0.783	0.784

Table 2 provides an exact numerical summary of the errors in the three discussed mass metrics. The presence of outliers seen in the previous graphs affected the mean and standard deviation. More robust location and dispersion statistics such as median, IQR (interquartile range), and MAD (median absolute deviation) indicate negligible prediction errors.

#### 3.2.2 Impurity and Degradant Analyses

**Figure 8:**
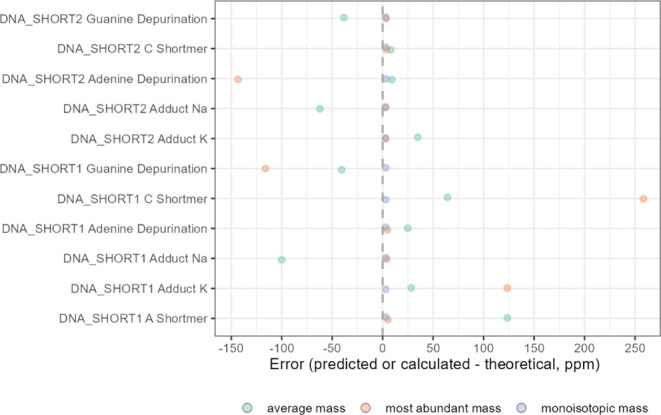
Errors in the average, most-abundant, and monoisotopic masses for selected modifications of DNA_SHORT1 and DNA_SHORT2 compounds.

Impurity and degradant annotation in experimental mass spectra is one of the essential activities during drug products’ characterization. For this reason, we screened the same data set to find certain selected impurities and degradants. To increase the signal-to-noise ratio in these inherently less abundant isotopic envelopes, we took the average over the retention times, giving rise to the average mass spectrum. After this step, isotope envelopes of the following “modifications” of the three DNA strands were extracted, namely, A shortmer, C shortmer, adenine and guanine depurination, and Na and K adducts. To rule out the potential impact of the incorrect most-abundant peak designation, we resorted to using the theoretical most-abundant peak masses of the 6 earlier mentioned “modifications” to predict their monoisotopic mass values (Figure 8). As a result, all monoisotopic mass predictions were very close to the theoretical values, in contrast to the other two mass metrics, the average mass and the most-abundant mass.

Furthermore, one can anticipate a wide array of other scenarios where the number of atoms of polyisotopic elements is altered or the compound itself contains polyisotopic elements which have not been considered in the training data set. For instance, it is quite common to use a phosphorothioate backbone for antisense applications because they are more resistant to nuclease degradation. To assess the model accuracy in such challenging settings, we conducted the following in silico validation exercise. We altered the atomic composition of the 10,000 test in silico-generated DNA oligonucleotides by introducing a certain number of times (e.g., 1, ···, 5 and 10 times) selected “artificial” or “biological” modifications. The BRAIN algorithm was employed once again to recompute theoretical isotope distributions, followed by monoisotopic mass prediction with MIND4OLIGOS. Note that this time we refrained from (1) adding noise on the BRAIN-computed peak intensities and (2) applying the most-abundant selection heuristic. Subsequently, we calculated the fraction of oligonucleotides where the forecasted monoisotopic mass closely matched the actual value (the “0 ppm” bin). Additionally, we determined the median accuracy within this bin and the range encompassing 95% of the sequences found in the bin. Since it is impossible to impart modifications on some short sequences, we report the frequency of such cases among the 10,000 molecules. Table 3 suggests that MIND4OLIGOS is relatively robust to various modification events, including depurination, shortmers, and mono- and polyisotope chemical element addition or removal. Incorporating 1, 2, or 3 sulfur atoms worsened but did not fully compromise our prediction strategy. For cases with more than three sulfur atoms, a different methodology would be needed.

**Table 3:** In Silico Incorporation of Selected Pharmaceutically Relevant Modifications to the 10,000 Test Oligonucleotides from the In Silico-Generated Data^a^

	modification	# of mod	%′0 ppm’	median	95% width	# missing counts
“artificial/chemical”	guanine depurination (–C_5_H_5_N_5_O + H_2_O)	1	94.80	–0.002	1.055	0
		2	94.79	0.000	1.054	0
		3	94.32	0.003	1.051	0
		4	93.57	0.005	1.055	0
		5	93.53	0.008	1.049	0
		10	89.89	0.024	1.500	1
	guanine shortmer (–C_11_H_14_N_5_O_7_P)	1	94.67	–0.005	1.059	0
		2	94.50	–0.006	1.060	0
		3	94.20	–0.005	1.059	2
		4	94.15	–0.004	1.062	3
		5	94.35	–0.004	1.061	4
	methylation (+CH_2_)	1	94.24	–0.005	1.059	0
		2	94.20	–0.004	1.059	0
		3	94.00	–0.004	1.058	0
		4	93.89	–0.003	1.057	0
		5	93.93	–0.003	1.057	0
		10	93.18	–0.001	1.054	0
**“**biological”	fluoride (–OH + F)	1	94.37	–0.005	1.060	0
		2	94.53	–0.005	1.060	0
		3	94.51	–0.005	1.060	0
		4	94.40	–0.004	1.060	0
		5	94.38	–0.004	1.060	0
		10	93.76	–0.003	1.057	0
	galnac conjugation (+C_87_H_140_O_41_N)	1	72.43	0.020	1.034	579
	oxygen-to-sulfur conversion (–O + S)	1	90.77	–0.023	1.085	0
		2	83.54	–0.040	1.121	0
		3	75.53	–0.056	1.155	0
		4	67.68	–0.073	1.192	0
		5	60.14	–0.089	1.235	0
		10	19.73	–0.173	1.414	0

a Each modification was added a number of times, as indicated by the third column. Columns 4–6 represent the proportion of monoisotopic mass estimates with the “0 Da” label, median ppm deviation, and the interval width containing 95% of the values with this label, respectively.

## 4 Conclusions

We have shown that the underlying idea incorporated in the original MIND method for proteins is generic and can be translated to other types of (bio)polymers, such as DNA/RNA oligonucleotides or else, as long as there are means to construct or retrieve a theoretical training data set for the molecules of interest. In contrast to the protein-centered approach, the overlap of residual lines’ mass domains affects only two consecutive lines (Figure 2), enabling the development of a more compact prediction method. The method’s good accuracy has been thoroughly demonstrated on an in silico data set and validated on experimental mass spectra. Even though MIND4OLIGOS was trained on unmodified oligonucleotide sequences, it is already capable of aptly handling selected modifications, as highlighted in the experimental and in silico analyses reported in the previous section. Future research will attempt at expanding the existing methodology in a way that would allow taking into account any user-specified modifications of oligonucleotides. Toward this goal, we will consider combining our previously developed POINTLESS4DNA methodology and signal convolution. Finally, we firmly believe that the proposed approach constitutes a valuable contribution toward the development of a general open-source analysis workflow aiming at automated annotation and quantitation of complex DNA/RNA mass spectra. The interested reader can test our methodology via an R Shiny app freely available at https://valkenborg-lab.shinyapps.io/mind4oligos/.

## Supporting Information Available

Estimated model coefficients of linear models for determining the monoisotopic mass of DNA and RNA oligonucleotides; applicability of MIND4OLIGOS based on the monoisotopic-to-most-abundant intensity ratio calculated for all in silico DNA oligonucleotides; quality of the training and test split up of the in silico DNA oligonucleotide data set; an example of an extracted isotope envelope with a missing monoisotopic peak; distribution of the difference between the intensity-weighted average masses and the most-abundant masses in the entire in silico DNA oligonucleotide data set; overview of the ion statistics and number of isotopic peaks across the “timsTOF” real-world data of the three selected oligonucleotides; simulation study to assess the accuracy of the most-abundant peak selection correction; effect of adding noise-to-peak intensities present in the in silico DNA oligonucleotide data set; validation of MIND4OLIGOS on the test part of the in silico RNA oligonucleotide data set; overview of the ion statistics and number of isotopic peaks in the real-world Synapt G2 HDMS QTOF data; errors in average, most-abundant masses, and monoisotopic mass predictions of the three DNA compounds—DNA_SHORT1, DNA_SHORT2, and DNA_LONG—measured with the Synapt G2 HDMS QTOF; and relationship between the errors in monoisotopic mass and errors in most-abundant mass (PDF)
