# Supplementary material for: MIND4OLIGOS: Determining the Monoisotopic Mass of Oligonucleotides Observed in High-Resolution Mass Spectrometry
Source: Anal Chem. 2024 May 28;96(23):9343–52. doi: 10.1021/acs.analchem.3c04351 (PMC11342294; doi:10.1021/acs.analchem.3c04351)
Supplement: Supplementary file 1 — ac3c04351_si_001.pdf [file ac3c04351_si_001.pdf]

Supporting Information MIND4OLIGOS: determining the monoisotopic mass of oligonucleotides observed in high-resolution mass spectrometry

Piotr Prostko[1], Piotr Radziński[2], Michał Ciach[2], Youzhong Liu[3], Michał Startek[2,4], Frederik Lermyte[5,6], Thomas De Vijlder[3], Anna Gambin[2], Simon Appeltans[1], Dirk Valkenborg[1*]

[1] Hasselt University, Faculty of Science, Data Science Institute, Interuniversity Institute for Biostatistics and statistical Bioinformatics, Center for Statistics, Agoralaan, Diepenbeek, BE 3500

[2] University of Warsaw, Institute of Informatics, Banacha 2, Warszawa, PL 02-097

[3] Johnson & Johnson Innovative Medicine, Therapeutics Development & Supply, Turnhoutseweg 30, Beerse, BE 2340

[4] University Medical Center of the Johannes Gutenberg University Mainz, Institute of Immunology, Mainz, Rheinland-Pfalz, DE 55131

[5] Technical University of Darmstadt, Department of Chemistry, Darmstadt, Hessen, DE 64289

[6] Technical University of Darmstadt, Centre for Synthetic Biology, Darmstadt, Hessen, DE 64289

[*]Email: dirk.valkenborg@uhasselt.be

Estimated model parameters

Table S1 Coefficients and mass ranges of the linear models for determining the monoisotopic mass of DNA oligonucleotides.

**Range most abundant mass (Da)**		**Intercept**	**Slope**
Model 1			
1463.242	30303.874	0.6624438	0.9995244
Model 2			
1463.242	2514.488	-0.6624438	0.0004755794
2514.488	4562.604	-1.6651512	0.0004755800
4562.604	6661.219	-2.6677113	0.0004755712
6661.219	8760.759	-3.6702752	0.0004755682

8760.759	10874.989	-4.6729650	0.0004755795
10874.989	12982.110	-5.6757768	0.0004755967
12982.110	15092.861	-6.6786484	0.0004756131
15092.861	17206.612	-7.6816303	0.0004756322
17206.612	19314.809	-8.6848041	0.0004756573
19314.809	21427.741	-9.6881583	0.0004756860
21427.741	23541.489	-10.6916671	0.0004757163
23541.489	25651.264	-11.6953531	0.0004757485
25651.264	27747.691	-12.7003241	0.0004758244
27747.691	30303.874	-13.7266085	0.0004766571

Table S2 Coefficients and mass ranges of the linear models for determining the monoisotopic mass of RNA oligonucleotides.

**Range most abundant mass (Da)**		**Intercept**	**Slope**
Model 1			
1543.217	31086.314	0.6771300	0.9995378
Model 2			
1543.217	2644.513	-0.677130	0.0004621878
2644.513	4751.652	-1.679791	0.0004621772
4751.652	6900.691	-2.682212	0.0004621541
6900.691	9065.444	-3.684638	0.0004621435
9065.444	11233.542	-4.687052	0.0004621363
11233.542	13402.604	-5.689425	0.0004621283
13402.604	15574.220	-6.691750	0.0004621194
15574.220	17746.725	-7.694018	0.0004621094
17746.725	19919.353	-8.696229	0.0004620987
19919.353	22091.896	-9.698388	0.0004620878
22091.896	24264.583	-10.700486	0.0004620762

24264.583	26437.309	-11.702526	0.0004620644
26437.309	28580.072	-12.708407	0.0004621964
28580.072	30918.106	-13.723912	0.0004626651
30918.106	31086.314	-14.714097	0.0004622735

Miscellaneous

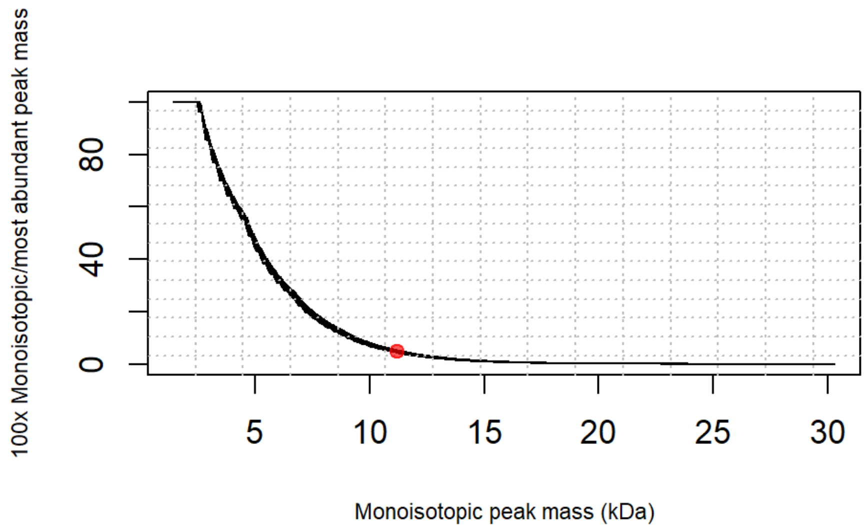

Figure S1 From approximately 11 181 Da onwards, the monoisotopic mass constitutes only 5% (or less) of the most abundant peak height in the theoretical DNA dataset. Hence, the likelihood of seeing the monoisotopic variant of moderately-sized oligonucleotides becomes relatively low. This observation highlights the importance of MIND4DNA in recovering that useful information.

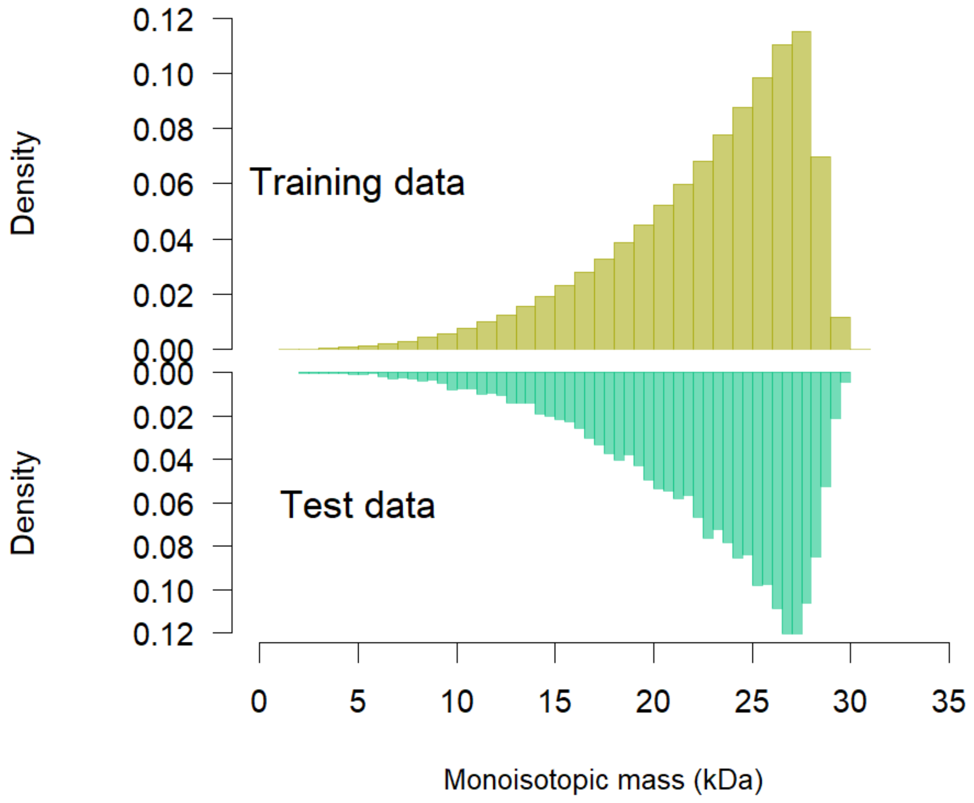

Figure S2 The distribution of molecules' monoisotopic masses in the training and test parts of the DNA theoretical dataset. Judging by the similarity of the two histograms, the sample of 10 000 molecules included in the test data is representative of the training part.

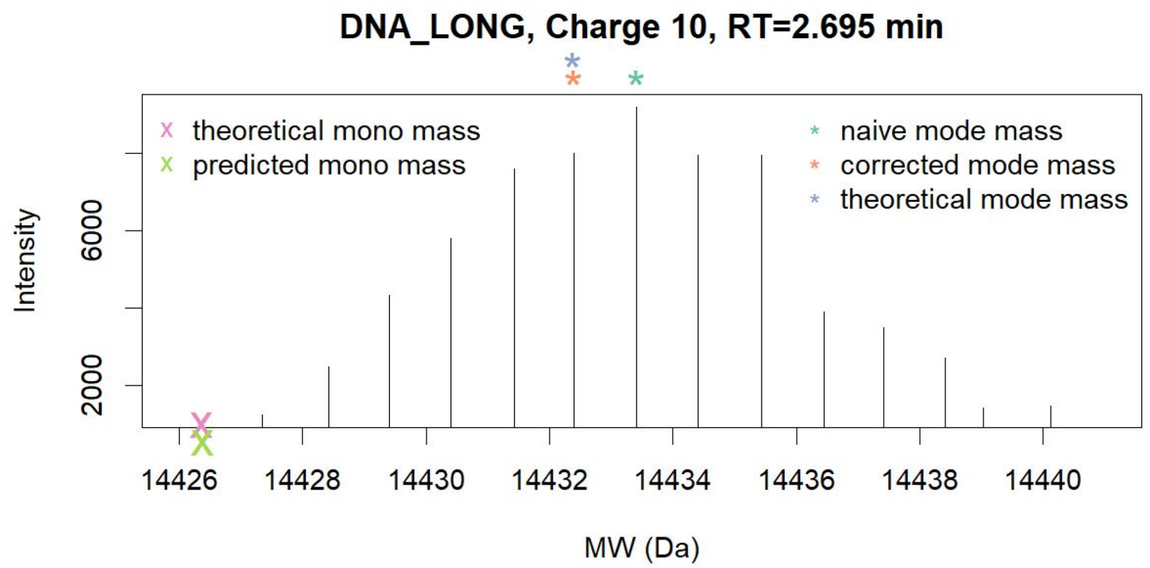

Figure S3 An example of an extracted isotope peak pattern of oligonucleotide DNA_LONG with missing monoisotopic peak. Due to low theoretical probability of the monoisotopic variant, our pre-processing workflow treated that peak as noise. Horizontal position of the three coloured stars indicate the most-abundant ("mode") mass. The most-abundant mass

selection heuristic explained in the main text recovered the correct value. This value supplied to MIND4OLIGOS resulted in a very accurate prediction of the monoisotopic peak location (the cross symbol in green).

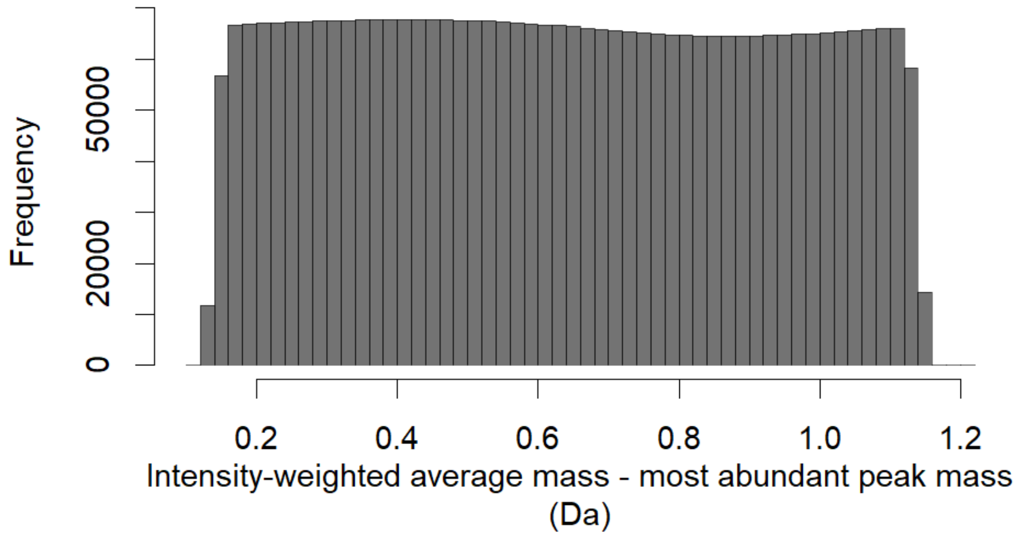

Figure S4 The distribution of the difference between the intensity-weighted average masses and most abundant peak masses computed on theoretical isotopic distribution. The fact that the majority of those differences are bounded by fixed values drives the most abundant peak mass selection heuristic briefly mentioned in the main text of this publication.

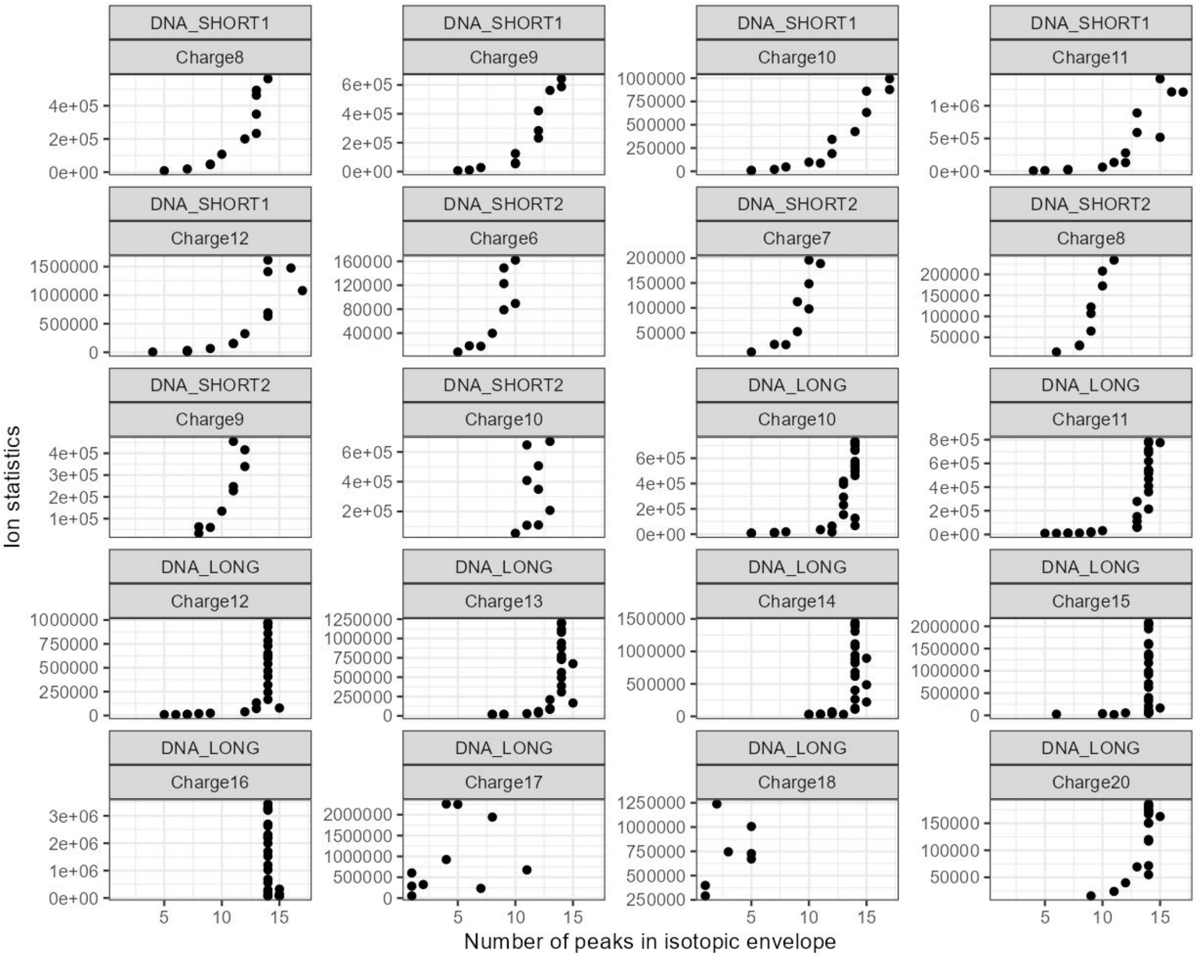

Figure S5 Overview of the ion statistics and number of isotopic peaks extracted from the timsTOF experimental data across different molecules and charges. Each point corresponds to an isotopic distribution retrieved from a mass scan taken at a certain retention time. Improvements in ion statistics increases the likelihood of detecting more experimental peaks.

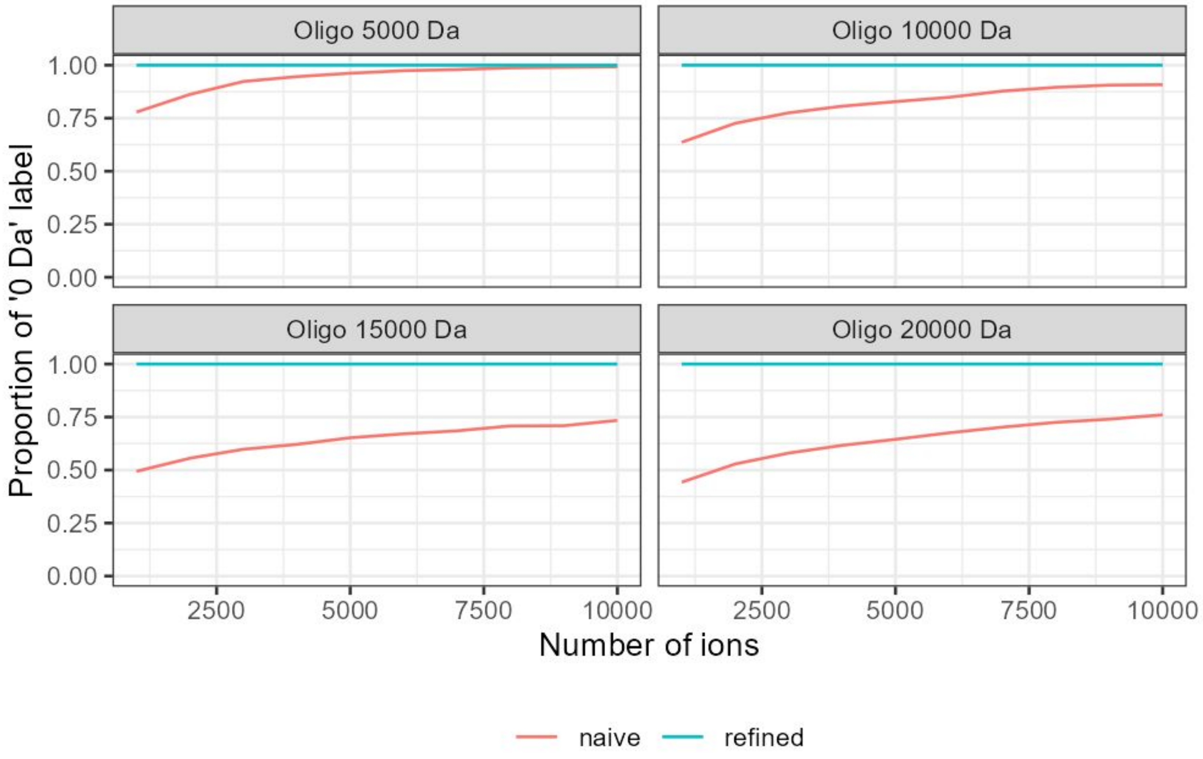

Figure S6 Accuracy of the most-abundant peak selection correction from the main text. We conducted a simulation of four theoretical oligonucleotides with a molecular weight of 5, 10, 15, and 20 kDa. Their theoretical isotope probabilities, computed with BRAIN, were supplied to a multinomial distribution with a total number of ions ranging from 1000 to 10 000. Then, we draw 5 000 times from such multinomial distributions and compare the index of truly most-abundant peak with the indices indicated by 'naïve' selection (red line) or the corrected selection (blue line). The y-axis corresponds to the proportion of cases where indices were in agreement. The correction consistently performs better than the naïve selection.

Peak intensity noise generation

Each isotopic peak I_k in the theoretical isotope distribution is replaced by independently drawn value from a uniform distribution U([I_k(1-c), I_k(1+c)]). We chose c equal to 5%, resulting in the simulated values centered at the original peak height with possible deviations up to 5% in both directions. No variability was added on peak masses. Figure S7A shows an overlay of an example theoretical isotope distribution with its corresponding noisy envelope. Adding noise in this particular exampled did not influence the position of the most abundant ion. Figure S7B presents the evolution of off-by-one errors in function of the total width of the noise-generating uniform distribution. Allowing for 15% one-sided deviation from the initial peak intensity resulted in a total off-by-one error of about 10%.

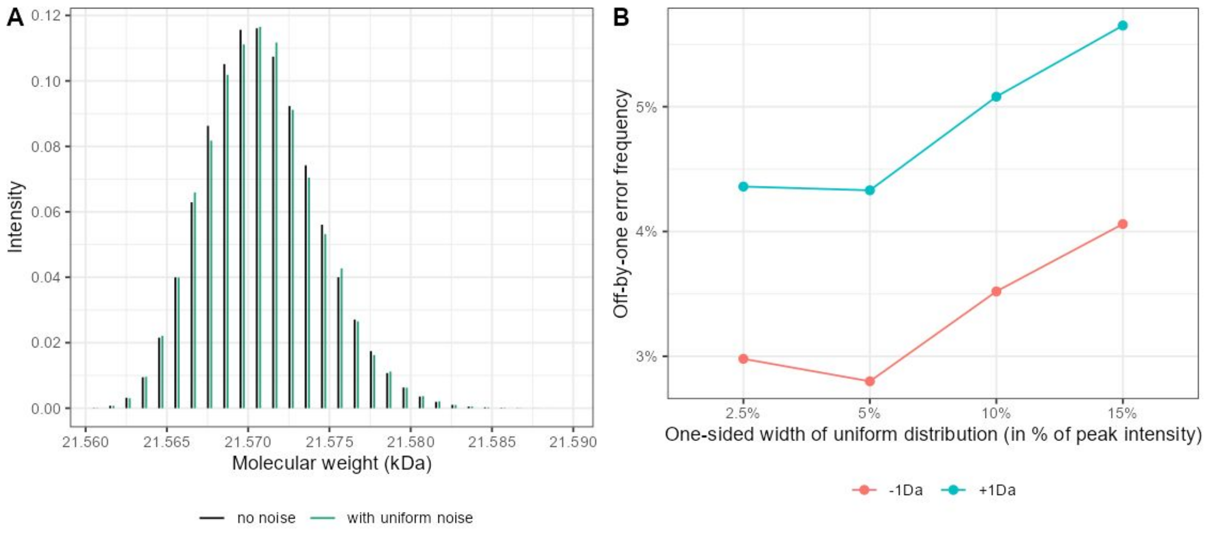

Figure S7 (A) The theoretical isotopic distribution (in black) of an DNA molecule overlayed with its noisy counterpart (in green). In this specific example, adding noise would not change the result of choosing the most abundant peak. To visually distinguish two envelopes, the green one was shifted by a small mass value. (B) The evolution of off-by-one errors over varying widths of the noise-generating uniform distribution.

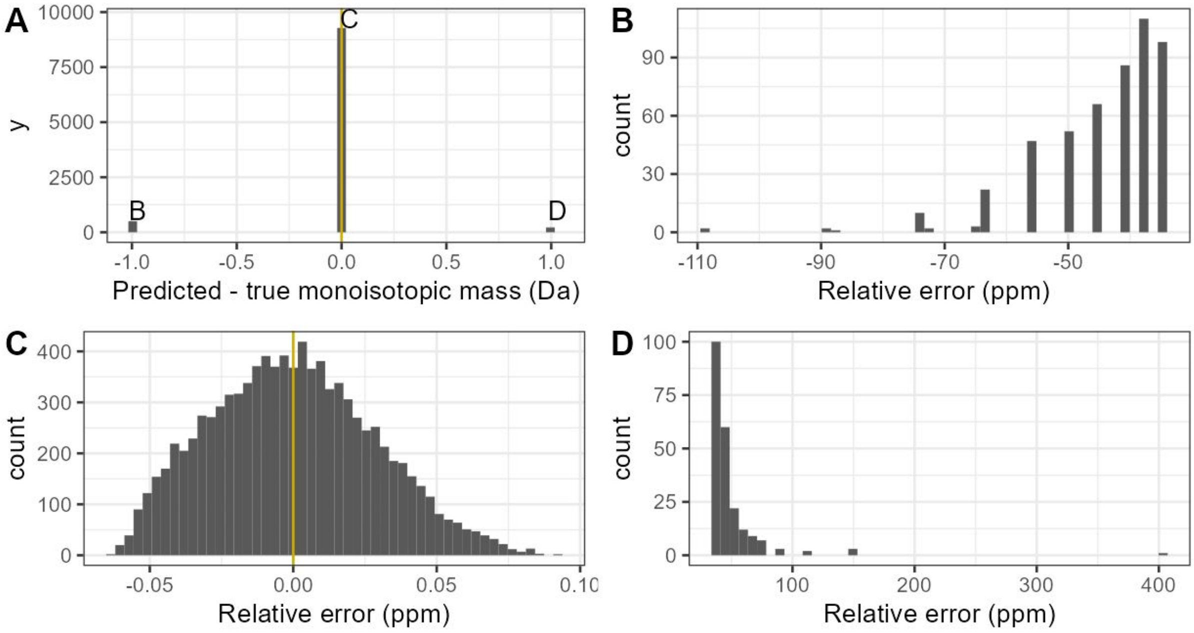

Figure S8 In silico validation of the data concerning 10 000 theoretical (unmodified) RNA sequences that were set apart from the training data. The isotopic peak heights were deliberately distorted by adding uniform noise. The resulting distribution of the off-by-one error (i.e., theoretical minus predicted monoisotopic mass value) is as follows: 92.8% accuracy, 2.19% and 5.01% of -1 Da and +1 Da errors, respectively. B-D) Relative prediction errors around the bars from panel A.

Validation on Synapt G2 HDMS QTOF data

The same oligonucleotides as explained in the main text of our manuscript were analysed with a slightly different LC-MS setup, leading to a different dataset. It can be noticed in the following graphs that this data acquisition resulted in isotope envelopes with lower intensities, represented by lower

number of peaks, and with minor mass miscalibration. Nevertheless, we still report the analysis results to demonstrate performance of MIND4OLIGOS applied to lower quality spectral data.

Experimental procedures

Measurements were acquired via LC-MS by injecting 10 µL of a ca. 0.1 mg/mL aqueous solution on an H-Class UPLC (Waters Inc., Antwerp, Belgium) coupled to a Synapt G2 HDMS QTOF mass spectrometer (Waters Inc.). Chromatographic separations were performed using an Acquity BEH 300 C18 column (150 × 2.1 mm, 1.7 µm particle size) (Waters Inc.). The column heater was kept at 75 ◦C and the flow rate was 0.25 mL/min. mobile phase A consisting of 7 mM triethylamine and 60 mM 1,1,1,3,3,3-Hexafluoro-2-propanol (HFIP) in water, and mobile phase B was a methanol–acetonitrile mixture (50/50, v/v). The gradient elution consisted of a linear gradient of 0% to 70% of eluent B in 30 min. followed by a washing step of 5 min at 70% mobile phase B. High resolution accurate mass data were acquired in negative ion mode using an electrospray ionisation source using a mass range of m/z 50 to m/z 2000 at a resolving power of approximately 12,000 (sensitivity mode, measured at m/z 1000). The following source conditions were applied: capillary voltage 2 kV, cone voltage 30 V, Extractor voltage 4 V, source temperature 120 ◦C, desolvation temperature 350 ◦C, cone gas flow 20 L/h.

MSConvert GUI [19] was applied to transform the raw vendor spectral files to mzXML format. Further, necessary processing steps were performed automatically in R programming language (v4.1.2) with an in-house script called OligoDistiller (https://github.com/daniellyz/OligoDistiller). To extract isotopic envelopes of one oligonucleotide from LC-MS data, we first selected all MS scans within fixed elution ranges. Subsequently, the charge state and neutral molecular weight (NMW) of each mass peak in the selected scans were computed by OligoDistiller. Isotopic envelope replicates of the same oligonucleotide were then collected from the. The NMW range was defined between the monoisotopic molecular weight (MMW) of an oligonucleotide and MMW + 15 Da.

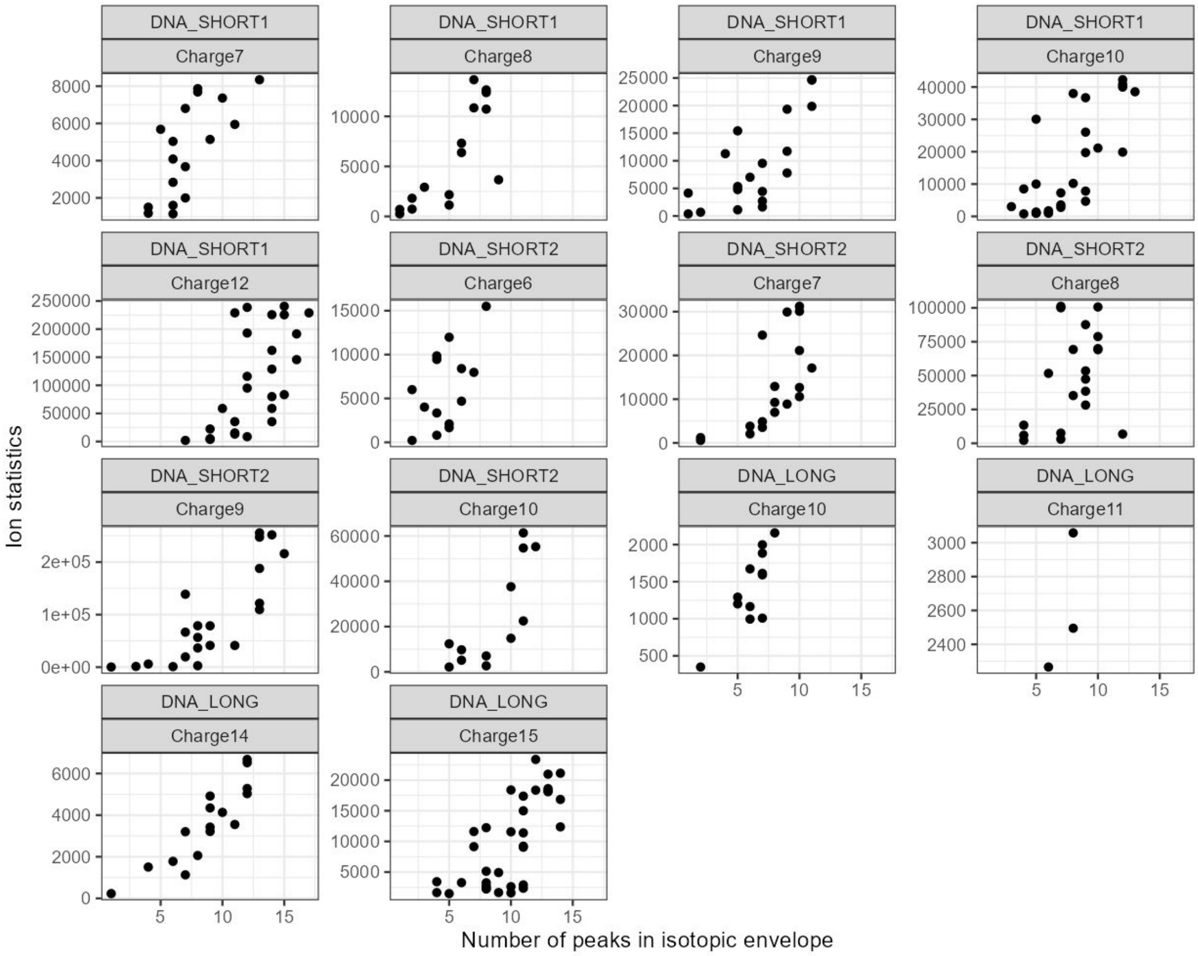

Figure S9 Overview of the ion statistics and number of isotopic peaks extracted from the Synapt G2 experimental data across different molecules and charges. Each point corresponds to an isotopic distribution retrieved from a mass scan taken at a certain retention time. Improvements in ion statistics increases the likelihood of detecting more experimental peaks. Applying our monoisotopic mass prediction method to very narrow and incomplete experimental envelopes may yield invalid outcomes.

Note that the dataset underlying Figure S10 and Figure S11 contained duplicated mass values and was analysed ‘as is’. This duplication was most likely caused by the digitisation step during data acquisition, making the mass values corresponding to the same peak identical across different retention times. However, the corresponding intensity values were distinct.

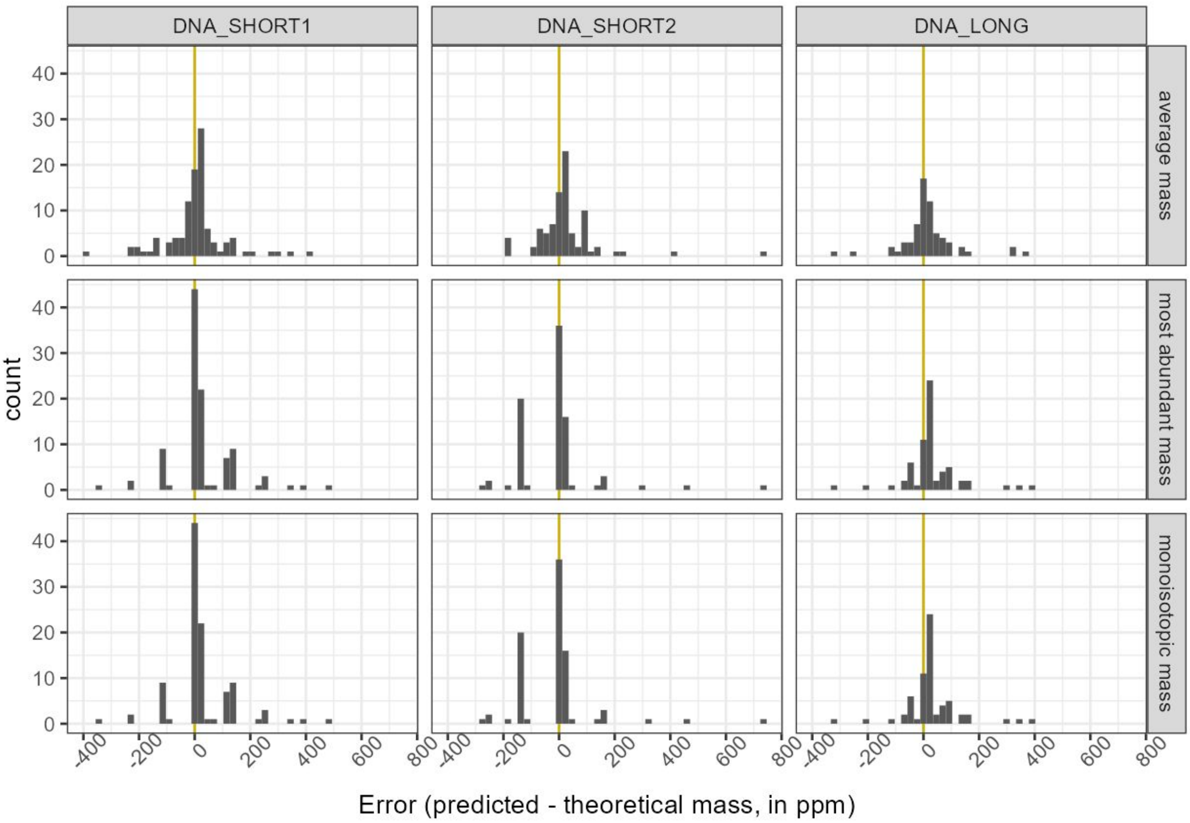

Figure S10 Errors in average, most-abundant masses and monoisotopic mass predictions of the three DNA compounds: DNA_SHORT1, DNA_SHORT2, and DNA_LONG, respectively. Do note that the error in most-abundant mass is the difference between the selected (using the heuristic) and theoretical most-abundant peak mass values. Mass scans acquired over a selected retention time range gave rise to multiple envelopes. Mass miscalibration observed in the most-abundant masses induced errors of approximately the same magnitude to the model predictions.

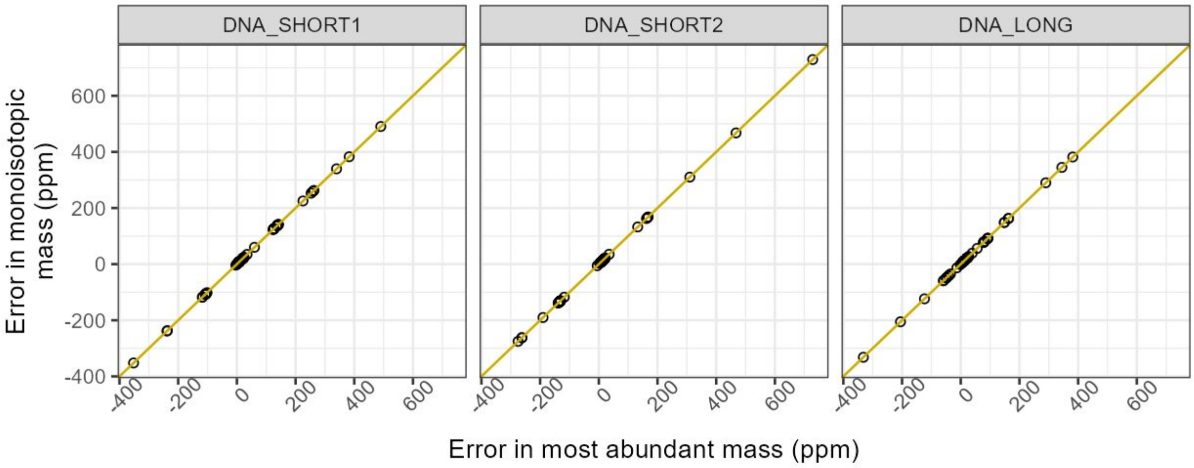

Figure S11 In this real-world validation dataset of three oligonucleotide strands, the errors in the monoisotopic mass prediction nearly perfectly correlated with the *errors* in the most-abundant masses as both *closely follow the gold identity line.*
